# Was Wuhan the early epicenter of the COVID-19 pandemic?—A critique

**DOI:** 10.1093/nsr/nwac287

**Published:** 2022-12-22

**Authors:** Yanan Cao, Lingling Chen, Hua Chen, Yupeng Cun, Xiaofeng Dai, Hongli Du, Feng Gao, Fengbiao Guo, Yalong Guo, Pei Hao, Shunmin He, Shunping He, XiongLei He, Zheng Hu, Boon-Peng Hoh, Xin Jin, Qian Jiang, Qinghua Jiang, Asifullah Khan, Hong-Zhi Kong, Jinchen Li, Shuai Cheng Li, Ying Li, Qiang Lin, Jianquan Liu, Qi Liu, Jian Lu, Xuemei Lu, Shujin Luo, Qinghua Nie, Zilong Qiu, Tieliu Shi, Xiaofeng Song, Jianzhong Su, Sheng-ce Tao, Chaolong Wang, Chuan-Chao Wang, Guo-Dong Wang, Jiguang Wang, Qi Wu, Shaoyuan Wu, Shuhua Xu, Yu Xue, Wenjun Yang, Zhaohui Yang, Kai Ye, Yuan-Nong Ye, Li Yu, Fangqing Zhao, Yiqiang Zhao, Weiwei Zhai, Dandan Zhang, Liye Zhang, Houfeng Zheng, Qi Zhou, Tianqi Zhu, Ya-ping Zhang

**Affiliations:** Ruijin Hospital, Shanghai Jiao Tong University, China; College of Life Science and Technology, Guangxi University, China; Beijing Institute of Genomics, Chinese Academy of Sciences, China; Children's Hospital of Chongqing Medical University, China; Wuxi School of Medicine, Jiangnan University, China; School of Biology and Biological Engineering, South China University of Technology, China; Department of Physics, School of Science, Tianjin University, China; School of Pharmaceutical Sciences, Wuhan University, China; Institute of Botany, Chinese Academy of Sciences, China; Institut Pasteur of Shanghai, Chinese Academy of Sciences, China; Institute of Biophysics, Chinese Academy of Sciences, China; Institute of Hydrobiology, Chinese Academy of Sciences, China; School of Life Sciences, Sun Yat-sen University, China; Institute of Synthetic Biology, Shenzhen Institute of Advanced Technology, Chinese Academy of Sciences, China; Faculty of Medicine and Health Sciences, University College Sedaya International, Malaysia; School of Medicine, South China University of Technology, China; Department of Medical Genetics, Capital Institute of Pediatrics, China; School of Life Science and Technology, Harbin Institute of Technology, China; Department of Biochemistry, Abdul Wali Khan University, Pakistan; Institute of Botany, Chinese Academy of Sciences, China; Xiangya Hospital, Central South University, China; Department of Computer Science, City University of Hong Kong, China; College of Life Science and Technology, Foshan University, China; South China Sea Institute of Oceanology, Chinese Academy of Sciences, China; College of Ecology, Lanzhou University, China; School of Life Sciences and Technology, Tongji University, China; School of Life Sciences, Peking University, China; Kunming Institute of Zoology, Chinese Academy of Sciences, China; School of Life Sciences, Peking University, China; College of Animal Science, South China Agricultural University, China; Institute of Neuroscience, Chinese Academy of Sciences, China; School of Life Sciences, East China Normal University, China; Nanjing University of Aeronautics and Astronautics, China; Wenzhou Institute, University of Chinese Academy of Sciences, China; Institute of Systems Biomedicine, Shanghai Jiao Tong University, China; Tongji Medical College, Huazhong University of Science and Technology, China; School of Life Sciences, Xiamen University, China; Kunming Institute of Zoology, Chinese Academy of Sciences, China; Division of Life Science and Department of Chemical and Biological Engineering, The Hong Kong University of Science and Technology, China; Institute of Microbiology, Chinese Academy of Sciences, China; School of Life Sciences, Jiangsu Normal University, China; School of Life Sciences, Fudan University, China; College of Life Science and Technology, Huazhong University of Science and Technology, China; International Center for Aging and Cancer, Hainan Medical University, China; Academy of Medical Science, Zhengzhou University, China; Faculty of Electronic and Information Engineering, Xi’an Jiaotong University, China; Bioinformatics and BioMedical Bigdata Mining Laboratory, School of Big Health, Guizhou Medical University, China; School of Life Sciences, Yunnan University, China; Beijing Institutes of Life Science, Chinese Academy of Sciences, China; College of Biological Sciences, China Agricultural University, China; Institute of Zoology, Chinese Academy of Sciences, China; Department of Pathology, and Department of Medical Oncology of the Second Affiliated Hospital, Zhejiang University School of Medicine, China; School of Life Science and Technology, ShanghaiTech University, China; School of Life Sciences, Westlake University, China; Life Sciences Institute, Zhejiang University, China; Academy of Mathematics and Systems Science, Chinese Academy of Sciences, China; Kunming Institute of Zoology, Chinese Academy of Sciences, China

Recently, Worobey *et al*. (2022) published a report entitled ‘The Huanan Seafood Wholesale Market in Wuhan was the early epicenter of the COVID-19 pandemic’ that succinctly summarizes their study [1]. A pre-print version of this study had earlier elicited a series of high-profile media coverages [2,3]. All these reports deliver a social-political message that the Huanan market is the epicenter of COVID-19.

At the outset, we shall clarify the meaning of ‘epicenter’ in the context of the pandemic. Epicenter, narrowly defined, is the point on the earth's surface directly above an earthquake. Hence, in this context, epicenter should be the place from which the pathogen spread globally to cause the pandemic. By this definition, what Worobey *et al*. identified is not the epicenter of the global pandemic for the obvious reason that the conclusion is based entirely on SARS-CoV-2 samples collected in Wuhan. Where else could they have pinpointed the epicenter with their samples? Had they used only virus data from the Antarctica, they would have concluded that some place on that continent is the epicenter. A relevant example is the island province across the Taiwan Strait. Studies that analyzed the virus data entirely from the island have also shown that the Taoyuan International Airport south of the city of Taipei is the ‘epicenter’. In short, what Worobey *et al*. (2022) show is that the early epidemic in Wuhan centered around its seafood market, analogous to the Taoyuan Airport ‘epicenter’ of Taiwan.

In a technical sense, Worobey *et al*.’s title is vague. Did they simply mean that the Huanan Market is the ‘epicenter’ of the early *local* epidemic in Wuhan? Nevertheless, the juxtaposition of ‘epicenter’ and ‘pandemic’ in their title must have meant the global epicenter. Indeed, the global media has read Worobey *et al*. to mean Wuhan, with its seafood market, as the epicenter of the entire pandemic [2–6].

Worobey *et al*. have failed to follow the standard practice of presenting a scientific report in the context of previous publications on the same subject. This is particularly important when the conclusion is diametrically opposed to those of previous publications. Worobey *et al*. should have compared their conclusions with studies that have more extensive geographic data. Furthermore, although the interest is in the early phase of the pandemic, it has been shown that samples from subsequent periods can be informative about the early phase by interpolation. After all, later samples are far more abundant and better organized when human societies became aware of the onset of epidemics.

We have compiled a set of reports on viral samples from wild animals [7–16] which, collectively, are far more global by geography than the report we critique here. Another set of diverse studies provides evidence that SARS-CoV-2 may have been spreading worldwide for weeks or even months prior to the epidemic in Wuhan in December 2019 [17–24]. Such reports have been brushed aside due to a mis-conception on the onset of epidemics.

The misconception is most explicitly stated in a recent news report [25] as follows: ‘the idea of a pandemic origin outside China is preposterous to many scientists, because there's simply no way SARS-CoV-2 could have come from some foreign place to Wuhan and triggered an explosive outbreak there without first racing through humans at the site of its origin’. In the absence of an evolutionary perspective, that human and chimpanzee could have a common ancestor would be equally ‘preposterous’. Cohen's other points including the integrity of Chinese scientists is not worthy of a response.

Using the branching process to model the evolution of epidemics, Ruan *et al*. [26,27] and Kucharski *et al*. [28] have shown that invasions into a new population could trigger local epidemics only sporadically in the early phase. Local epidemics may even reach an alarming level of infections before fading out on its own. In this sense, there may be many earlier local epidemics (or endemics) that rise and fall before the eventual success of global spread from the true epicenter of the pandemic. The many reports of local infections prior to the global pandemic could be such a manifestation [17–24].

Ruan *et al*.’s [29] title ‘The twin-beginnings of COVID-19 in Asia and Europe—one prevails quickly’ may be a most explicit analysis of multiple early events. Their analysis has corroborated the earlier sampling results from the Lombardy region of Italy [20,21]. All these studies have concluded that Wuhan is not likely to be the epicenter of the COVID-19 pandemic. Finally, however the critiques and debates in the scientific community may resolve the issue, it is unfortunate that the pre-print version of Worobey *et al*. has attained unwarranted publicity on a subject of enormous social and political implication. The pre-print platform should be used by the scientific community to speed up exchanges, rather than by investigators to influence the societies at large before being debated among scientists.
